# Advances in hyaluronic acid hydrogel for meniscus repair

**DOI:** 10.3389/fbioe.2025.1639034

**Published:** 2025-07-21

**Authors:** Guangxin Wang, Xiao Jun liu, Xin-an Zhang, Mingjie Hu

**Affiliations:** ^1^ Department of Orthopedics, the Fourth People’s Hospital of Shenyang, Shenyang, China; ^2^ Department of Orthopedics, Qianjiang People’s Hospital, Qianjiang, China; ^3^ College of Exercise and Health, Shenyang Sport University, Shenyang, China

**Keywords:** meniscus injury, hyaluronic acid hydrogel, meniscus repair, cell carrier, tissue engineering, regenerative medicine

## Abstract

The meniscus, an important fibrocartilaginous structure within the knee joint, plays essential roles in shock absorption, joint stabilization, and the optimization of mechanical force transmission. Meniscus injuries are common among athletes, middle-aged and elderly individuals, and those engaged in heavy physical labor. Both conservative and surgical treatments currently have limitations, making it difficult to achieve complete repair and functional restoration of meniscal tissue. In recent years, hyaluronic acid (HA) hydrogel has shown broad potential in meniscus repair due to its excellent biocompatibility and biodegradability. As a cell carrier, this material not only promotes cell migration, proliferation, and differentiation but also mimics the biomechanical properties of the meniscus, regulates the inflammatory environment, and facilitates angiogenesis, thereby creating favorable conditions for tissue regeneration. This review summarizes the mechanisms, current applications, and research advances of HA hydrogel in meniscus repair, aiming to provide new theoretical foundations and technical support for meniscus injury treatment and to promote clinical translation and development in this field.

## 1 Introduction

**FIGURE 1:**
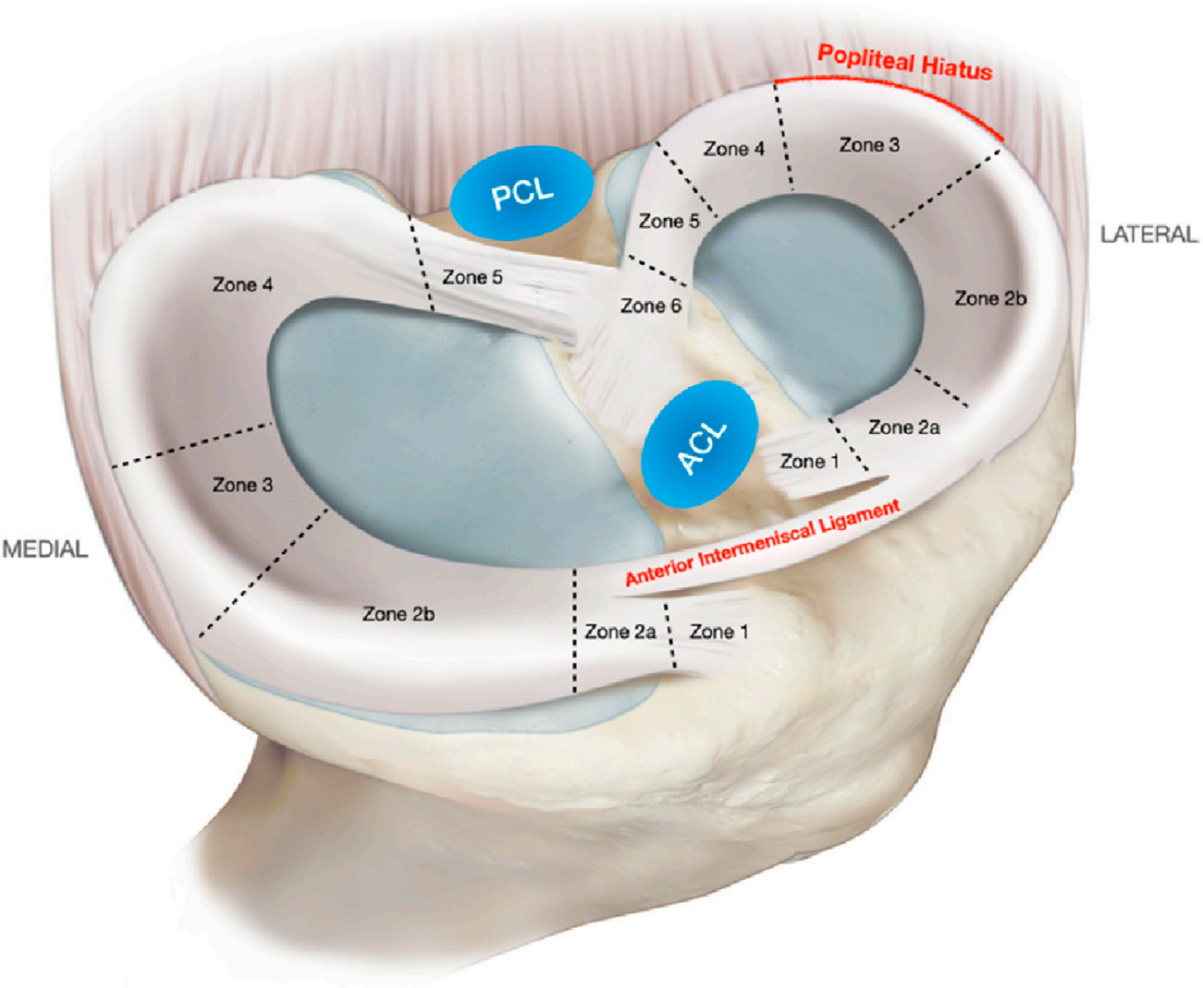
Medial and lateral meniscus zones and relevant anatomical relations. ACL, anterior cruciate ligament; PCL, posterior cruciate ligament. Reproduced with permission from ref Mameri et al. (2022). Copyright ^©^ 2022, The Author(s).

The meniscus (Figure 1) is one of the most important components of the human knee joint (Pasiński et al., 2023), playing a critical role in shock absorption, enhancing joint stability, lubricating joint surfaces, and optimizing force transmission (Mameri et al., 2022; Adams et al., 2021). Meniscus injury remains a pervasive and costly clinical challenge, burden increasingly amplified by aging global populations experiencing natural tissue degeneration and rising rates of sports participation and associated trauma, together driving significant and escalating demand for effective interventions (Adams et al., 2021). Currently, meniscus injury treatments primarily include conservative and surgical approaches (Giuffrida et al., 2020), against this backdrop, conventional treatments present persistent limitations that fail to adequately address the scope of the problem. Conservative approaches like physical therapy offer symptomatic relief but fundamentally cannot initiate biological repair of torn tissue (Sari and Kurniawati, 2022). Surgical options represent a historical compromise: partial meniscectomy removes damaged sections to alleviate pain (Wang H. et al., 2024a) but inevitably diminishes the meniscus’s vital biomechanical functions, inevitably accelerating joint degeneration and osteoarthritis risk (McDermott and Amis, 2006). While suturing aims for anatomical repair, its utility is severely constrained by factors like tear location (particularly in the poorly vascularized inner zones), chronicity, and patient age, leading to variable success rates, complex recovery periods, and procedural risks like infection and non-union (Popper et al., 2023); crucially, even successful repair often cannot fully restore the native tissue’s complex structure and function, representing a significant therapeutic gap. Motivated precisely by these long-standing inadequacies–the unmet need to achieve biological healing of tears (especially in challenging zones) and restore functional, protective meniscus tissue to halt the cascade towards osteoarthritis-research has increasingly focused on tissue engineering and regenerative medicine strategies (Bian et al., 2022; Lombardo et al., 2021). Stem cell therapy and novel biomaterials hold theoretical promise. However, practical translation faces formidable barriers, including identifying reliable cell sources, designing scaffolds that effectively mimic the anisotropic biomechanical properties of the native meniscus while supporting cellular integration and tissue formation, and ensuring long-term safety and efficacy (Li et al., 2023); It is within this specific context of unresolved needs and defined knowledge gaps that Hyaluronic Acid (HA) hydrogel has emerged as a compelling solution (Li et al., 2019). Its inherent high biocompatibility and biodegradability provide a foundation. Functionally, HA hydrogel offers potential as an injectable cell or bioactive factor carrier, fostering cell migration, proliferation, and differentiation directly at the injury site (Gupta et al., 2019; Teng et al., 2021). Critically, it represents a platform uniquely positioned to address the core limitations identified above: by potentially matching biomechanical cues, regulating the local inflammatory milieu, encouraging controlled vascular in-growth specifically where beneficial, and providing structural support, HA hydrogel aims to create the precisely tailored microenvironment necessary for functional meniscus regeneration (Li et al., 2024).

This review will summarize research advances in HA hydrogel for meniscus repair. By examining its mechanism of action, application value, and current research status, it aims to provide a new theoretical foundation and technical pathway for meniscus injury treatment. Moreover, it will guide clinical applications and future research, holding significant importance in advancing meniscus repair therapies.

## 2 HA hydrogel

HA (HA), as a naturally occurring polysaccharide, has garnered significant attention in the biomedical field due to its unique structure and diverse functions (Yasin et al., 2022). From its chemical structure and biocompatibility to its mechanical properties, the characteristics of HA endow it with great potential for applications in tissue engineering (Tsanaktsidou et al., 2022), drug delivery (Buckley et al., 2022), and joint lubrication (Su et al., 2023). The following sections provide a detailed overview of the structure and properties of HA from three perspectives: chemical structure, biocompatibility and biodegradability, as well as mechanical properties and viscoelasticity.

### 2.1 Structure and properties of HA

**FIGURE 2:**
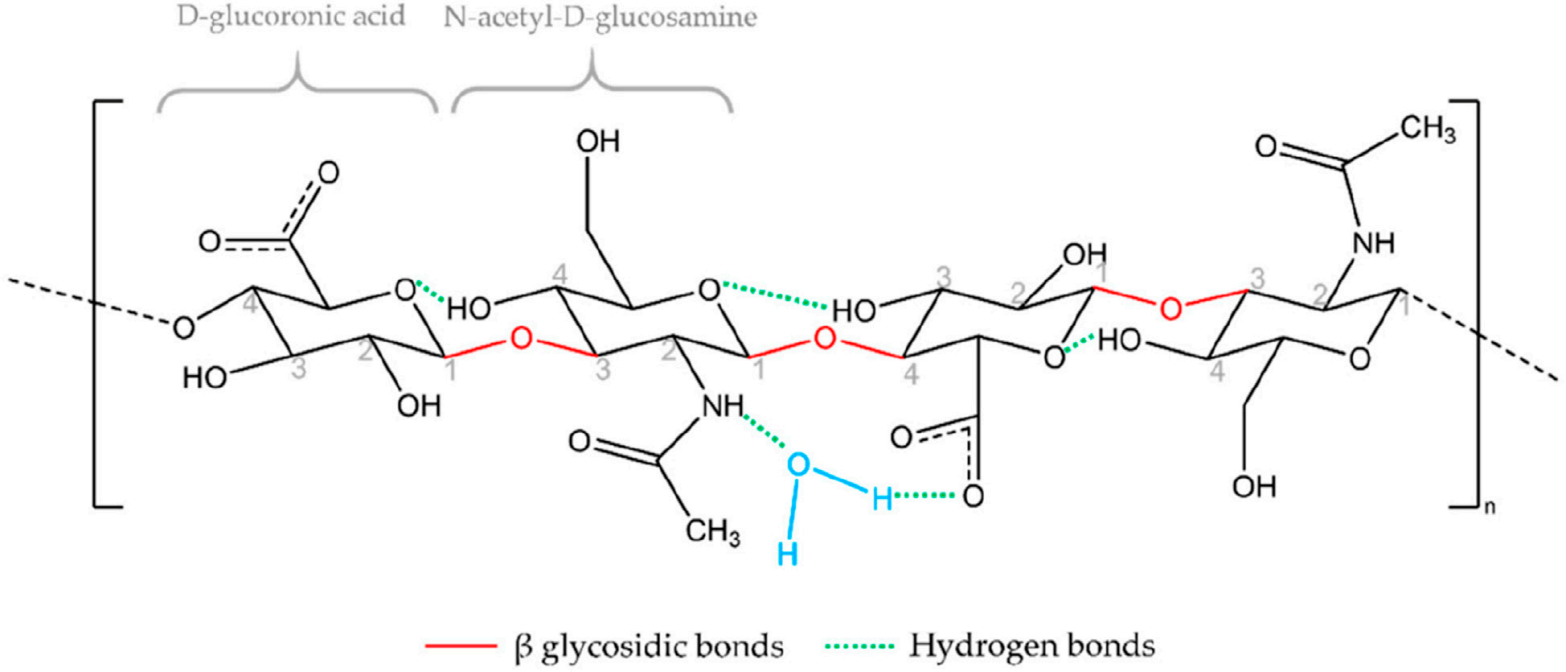
Chemical structure of HA. Reproduced with permission from ref Marinho et al. (2021). Copyright ^©^ 2021, The Author(s).

HA is a naturally occurring linear polysaccharide, whose chemical structure (Figure 2) consists of repeating disaccharide units of D-glucuronic acid and N-acetylglucosamine linked by β-1,3 and β-1,4 glycosidic bonds (Marinho et al., 2021). This structure is simple yet highly regular, with molecular chains rich in hydroxyl and carboxyl groups, endowing HA with pronounced hydrophilicity and a strong negative charge (Laffleur et al., 2025). HA forms hydrogen bonds with water molecules, creating a highly hydrated network structure that results in excellent water retention capacity and lubricating properties (Yasin et al., 2022). In addition, the molecular weight of HA spans a broad range, from several thousand to several million Daltons, and HA of different molecular weights exhibits significant differences in biological functions and applications. High molecular weight HA typically provides greater water retention and viscoelasticity, while low molecular weight HA more readily penetrates tissues to exert specific biological effects (Jabbari et al., 2023). This structural diversity gives HA broad application potential in the biomedical field.

Due to its natural presence in human tissues, such as synovial fluid, skin, and the vitreous body-HA exhibits excellent biocompatibility (Valachová and Šoltés, 2021). It does not trigger immune rejection and can interact with cell surface receptors (such as CD44 and RHAMM), participating in various biological processes including cell migration, proliferation, and differentiation (Rosales et al., 2024). *In vivo*, HA can be degraded by hyaluronidase and reactive oxygen species (Weber et al., 2019; Šoltés et al., 2006), with its degradation rate depending on molecular weight, degree of crosslinking, and local enzymatic activity. This tunable degradability provides HA with broad application prospects in tissue engineering and drug delivery.

The mechanical properties and viscoelasticity of HA are among its most important characteristics. Due to the flexibility of its polymer chains and its highly hydrated nature, HA exhibits pronounced viscoelastic behavior in solution (Fundarò et al., 2022). Its viscosity increases significantly with higher concentration and molecular weight, enabling it to form highly viscous, gel-like structures. This viscoelasticity allows HA to serve as a lubricant and shock absorber in synovial fluid, reducing friction and wear of articular cartilage (Zhang and Christopher, 2015). Additionally, the mechanical properties of HA can be tuned through chemical crosslinking or by combining it with other biomaterials to meet the requirements of various applications (Khanlari et al., 2015; Kong et al., 2020). Crosslinked HA hydrogels possess greater mechanical strength and stability, allowing them to withstand certain mechanical loads in the body while retaining their bioactivity and degradability (Khunmanee et al., 2017). For example, in joint injections, crosslinked HA can provide prolonged lubrication and help delay joint degeneration (Qiu et al., 2023).

### 2.2 Mechanism of HA in the meniscal repair

The underlying mechanism by which hyaluronic acid (HA) facilitates meniscal healing has been increasingly elucidated. Research demonstrates that HA promotes human meniscus regeneration, primarily through inhibiting apoptosis, enhancing cell migration, and accelerating cell proliferation. Murakami et al. (2019) specifically examined the effects of HA on prostaglandin E2 (PGE2)-induced apoptosis and gene expression in meniscus cells. Their findings confirmed that HA concentration-dependently increases both cell migration and proliferation in both the inner and outer regions of the meniscus. Crucially, these beneficial effects were blocked by an anti-CD44 antibody, indicating the essential role of the CD44 receptor. Further mechanistic insight revealed that HA activates the phosphatidylinositol 3-kinase (PI3K) and mitogen-activated protein kinase (MAPK) pathways, activation which was also prevented by anti-CD44 antibody treatment. This strongly suggests HA acts *via* the CD44 receptor to stimulate the PI3K/MAPK pathway. Additionally, HA was found to upregulate the mRNA levels of Collagen Type II Alpha 1 Chain (COL2A1) and Aggrecan (ACAN) specifically in inner meniscus cells. Based on these results, Murakami et al. concluded that HA holds promise for clinical application in managing meniscal injuries. Furthermore, the well-established anti-inflammatory and analgesic properties of HA (Salathia et al., 2023) are also considered critical for supporting overall meniscus repair.

### 2.3 Preparation methods of HA hydrogels

The preparation methods of HA hydrogels mainly include chemical crosslinking, physical crosslinking, and composite preparation with other biomaterials. Each method has its own characteristics and can be used to adjust the mechanical properties, degradation rate, and biological activity of the hydrogel according to specific application requirements. By selecting different preparation methods, it is possible to meet the needs of various application scenarios ranging from tissue engineering to drug delivery.

#### 2.3.1 Chemical crosslinking

Chemical crosslinking involves the formation of covalent bonds between HA molecular chains through chemical reactions (Wang et al., 2022), thereby constructing a stable three-dimensional network structure. Commonly used chemical crosslinking agents include Glutaraldehyde (GTA), divinyl sulfone (DVS), adipic acid dihydrazide (ADH), and carbodiimides (EDC). These crosslinking agents react with hydroxyl or carboxyl groups on HA molecular chains to form stable crosslinking points (Luo et al., 2023; Khunmanee et al., 2017). Hydrogels prepared by chemical crosslinking possess higher mechanical strength and stability, allowing them to withstand certain mechanical loads *in vivo* (Fuchs et al., 2020). Furthermore, the degree of crosslinking can be precisely controlled by adjusting the concentration of crosslinking agents and reaction conditions, thereby regulating the degradation rate of the hydrogel. For example, by increasing the concentration of crosslinking agents or extending the reaction time, the crosslinking density of the hydrogel can be enhanced (Nasution et al., 2022), improving its mechanical strength and stability. However, chemical crosslinking processes may introduce residual crosslinking agents (Sapuła et al., 2023), necessitating rigorous purification to ensure biosafety. To reduce the residual crosslinking agents, mild crosslinking conditions can be adopted or more biocompatible crosslinking agents can be used, such as natural crosslinking agents (like genipin) or photo-crosslinking agents (like methacrylates) (Yang et al., 2023). Additionally, chemical crosslinking methods can be combined with other techniques, such as microfluidic technology, to prepare hydrogels with complex structures and functions (Chen et al., 2021).

#### 2.3.2 Physical crosslinking

Physical interactions, including electrostatic interactions, chain entanglement, hydrophobic self-assembly, hydrophobic/hydrophilic interactions, and hydrogen bonds, have been developed as common crosslinking strategies for the preparation of HA-based hydrogels (Faivre et al., 2021). Physical crosslinking normally leads to a rapid polymerization behavior under relatively mild conditions and needs no toxic crosslinkers or catalysts, decreasing potential cytotoxicity (Huang et al., 2017; Baek et al., 2018). However, physically crosslinked hydrogels typically have lower mechanical strength and poorer stability (Xu et al., 2021), which may limit their use in certain high-load applications.

#### 2.3.3 Composite preparation with other biomaterials

To enhance the properties of HA hydrogels, they are often combined with other biomaterials such as collagen, chitosan (Wang et al., 2024b). Composite hydrogels integrate the excellent biocompatibility and lubricating properties of HA with the mechanical strength and functional characteristics of other materials. For example, combining HA with collagen can improve the hydrogel’s cell adhesion and tissue regeneration capabilities (Hwang et al., 2022); blending with chitosan can enhance the hydrogel’s antibacterial properties (Bai et al., 2023). The design of composite hydrogels can be flexibly adjusted according to the requirements of specific applications, allowing for precise control of their mechanical properties, degradation rate, and bioactivity to meet diverse clinical needs. For instance, in tissue engineering, composite hydrogels can serve as scaffold materials to support cell growth and tissue regeneration (Wang et al., 2021; Liang et al., 2023); in drug delivery, composite hydrogels can act as carriers, enabling sustained and targeted release of therapeutic agents (Svarca et al., 2022; Rong et al., 2023).

### 2.4 Intelligent responsive HA hydrogels

Intelligent Responsive HA Hydrogels are advanced biomaterials engineered by combining the inherent, advantageous properties of the natural biopolymer HA, namely, its outstanding biocompatibility, biodegradability, and ease of chemical modification with functional groups that exhibit sensitivity to external physical or chemical stimuli [e.g., pH (Qian et al., 2019), temperature (Ha et al., 2006), enzymes (Tam et al., 2017), light (Tam et al., 2017; Zhao et al., 2020), glucose (Xu et al., 2022), redox status (Zhang et al., 2017; Huang et al., 2022; Shi et al., 2024)]. This integration enables the dynamic environmental responsiveness and significant physicochemical property transitions characteristic of these materials; their sensitivities can be strategically combined to achieve multi-responsiveness tailored for precise interaction within complex pathological microenvironments. Demonstrating significant potential in medical applications, these smart materials facilitate on-demand drug release within lesion sites (such as acidic (Qian et al., 2019), highly reducing (Zhang et al., 2017), or enzymatic tumor microenvironments (Yang et al., 2015)) or *via* external triggers (e.g., light/heat) (Ha et al., 2006; Xu et al., 2020) in drug delivery, significantly improving targetability and minimizing side effects. Within tissue engineering, serving as intelligent scaffolds, they regulate cellular behavior and guide tissue regeneration through responsive degradation and signaling factor release (Lam et al., 2014). Furthermore, in wound repair, they not only manage a moist environment but also respond to variations in exudate pH or infection-related protease levels to release antibacterial or anti-inflammatory agents, promoting healing (Mohammed et al., 2022). Despite facing challenges concerning *in vivo* enzymatic stability, manufacturing standardization, complex system characterization, and precise *in vivo* control, intelligent responsive HA hydrogels, leveraging their engineerable dynamic responsiveness, multifunctional integration capacity, and HA’s inherent biological merits, are emerging as key Frontier materials driving advancements in precision drug delivery, intelligent tissue regeneration, and diagnostic/therapeutic integration.

## 3 Preclinical studies

**FIGURE 3:**
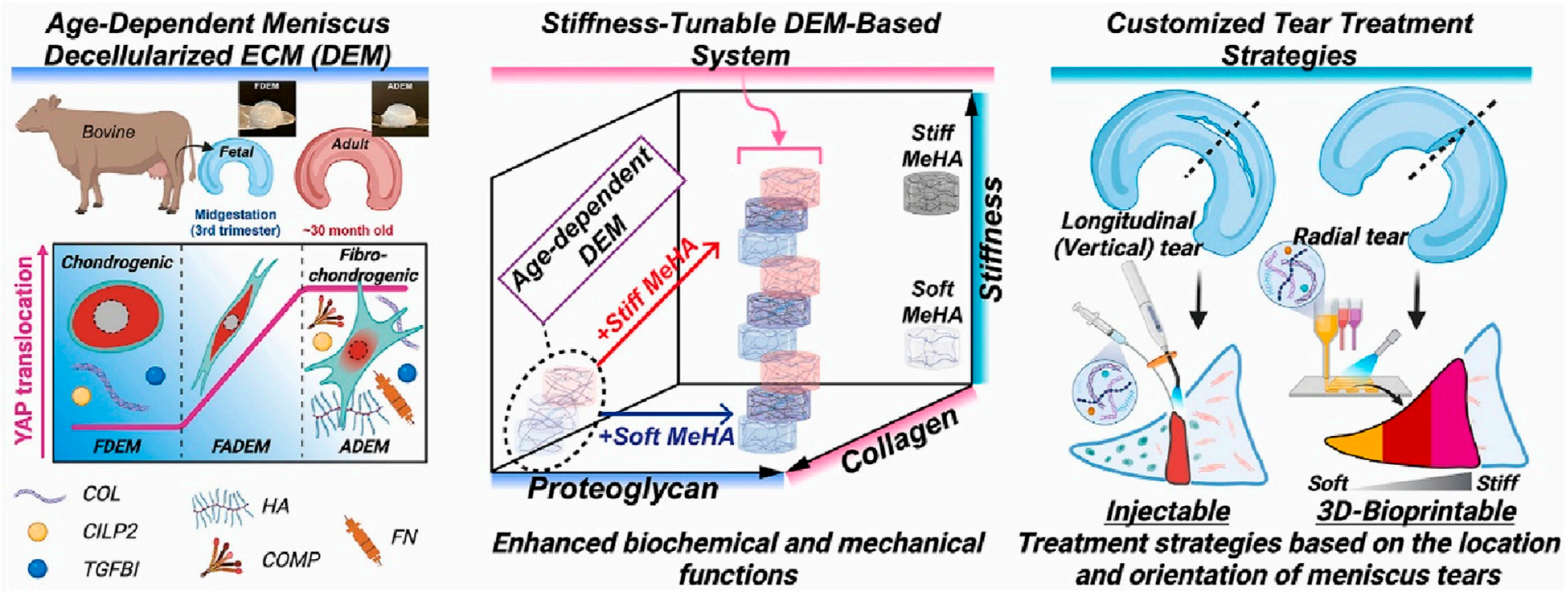
Precision repair of zone-specific meniscal injuries using decellularized extracellular matrix-based hydrogel system incorporating methacrylated hyaluronic acid. Reproduced with permission from ref Lee et al. (2025). Copyright ^©^ 2025, The Author(s).

**FIGURE 4:**
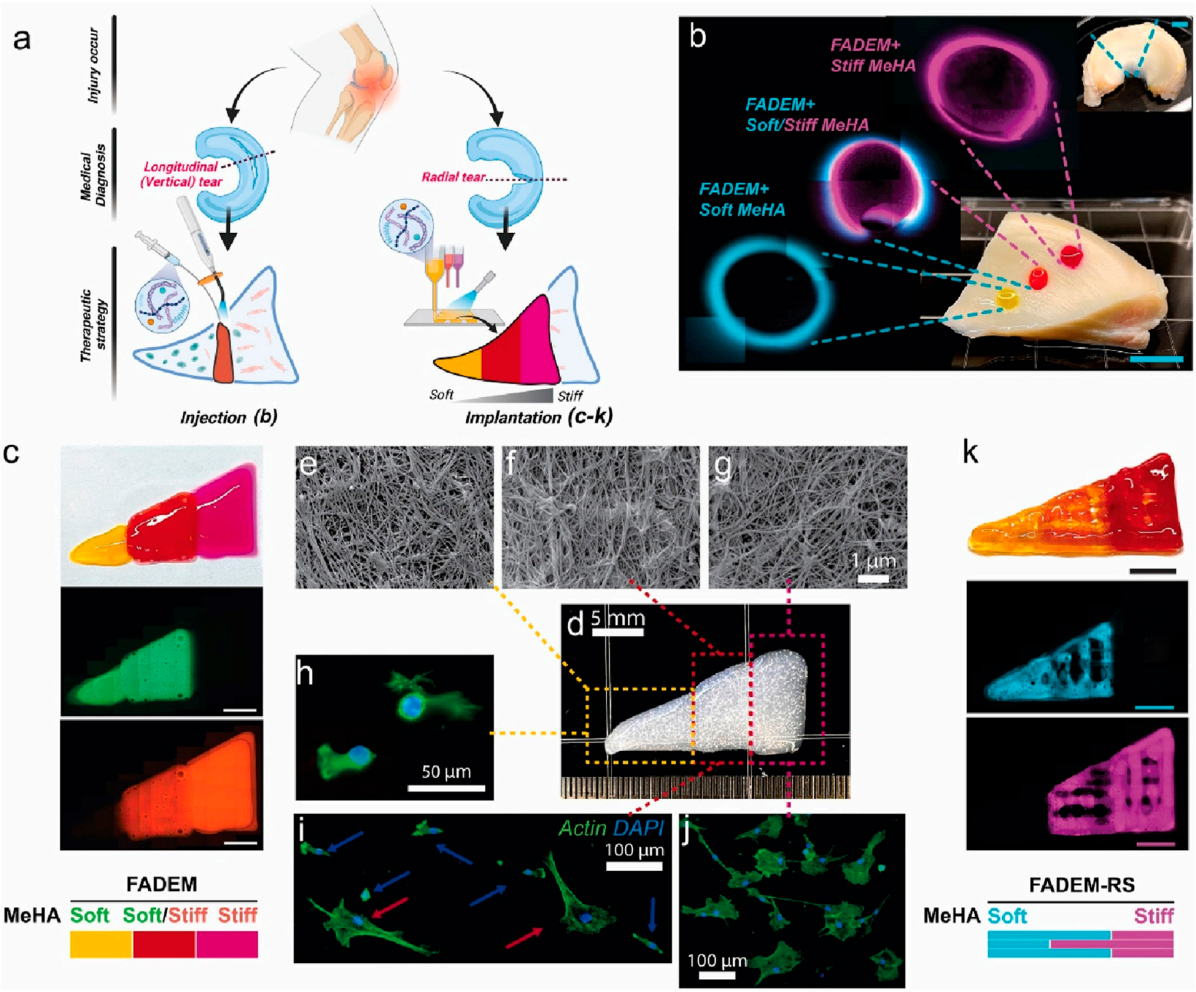
Targeted Meniscus Repair Using Stiffness-Tunable DEM-Based Hydrogels. **(a)** Illustration of the application of the system. Injection method: **(b)** injected stiffness-modulated FADEM-based hydrogel in defects in different meniscus zones (scale bar: 5 mm). Implantation method using 3D printed hybrid constructs: **(c)** images of a bioprinted stiffness-modulated FADEM-based hydrogel. **(e–g)** Representative FE-SEM images of fibrous structure of printed FADEM-based hydrogels. **(h–j)** F-actin staining of MSCs cultured on the zone-dependent printed FADEM-based hydrogels. **(k)** 3D printed lattices of zone-specific FADEM-based hydrogels with the addition of ruthenium/sodium persulfate (scale bars: 5 mm). Reproduced with permission from ref Lee et al. (2025). Copyright ^©^ 2025, The Author(s).

HA hydrogels demonstrate diverse application prospects in the field of meniscal injury repair. For instance, a decellularized extracellular matrix (dECM)-based hydrogel system incorporating methacrylated hyaluronic acid (meHA) (Figure 3) has been developed for the precision repair of meniscal injuries (Lee et al., 2025). *In vivo* tests confirmed the biocompatibility of hydrogels and their integration with native meniscus tissues. Furthermore, advanced 3D bioprinting techniques enabled the fabrication of hybrid hydrogels with biomaterial and mechanical gradients, effectively emulating the zonal properties of meniscus tissue and enhancing cell integration (Figure 4). This study represents a significant advance in meniscus tissue engineering, providing a promising platform for customized regenerative therapies across a range of heterogeneous fibrous connective tissues. This innovative system represents a significant advancement in meniscal repair, effectively addressing the challenges posed by heterogeneous meniscal injuries. The study points out that traditional repair strategies often fail to replicate the complex zonal characteristics of meniscal tissue, resulting in suboptimal healing outcomes. However, although this novel hydrogel system shows promise for precision repair, the research did not investigate the potential long-term effects or durability of engineered tissues in clinical applications, which may constrain the translational relevance of the findings.

In exploring microenvironmental regulation mechanisms, the pentenoic acid-functionalized hyaluronic acid (PHA) hydrogel system provides a platform for investigating mechanotransduction cues in meniscal fibrochondrocytes (MFCs) within the context of meniscal injury repair (Burkey et al., 2023). By modulating the degree of substitution (DoS) of reactive alkene groups, researchers achieved tunable crosslinking density and physical properties (including hydrogel crosslinking density, swelling ratio, and compressive modulus), which subsequently influenced MFC morphology and promoted regenerative phenotypes. This approach highlights the potential of PHA hydrogels for optimizing cellular microenvironments to enhance post-injury meniscal tissue regeneration. The study also indicates that while the developed HA hydrogel system possesses adjustable crosslinked network properties, its degradation rate is inversely correlated with DoS levels (lower DoS = faster degradation), which may compromise long-term structural integrity in physiological environments. Crucially, regulating the surface elastic modulus of these hydrogels was proven to modulate MFC morphology, suggesting that softer hydrogels preferentially induce an inner meniscus-like phenotype. However, the engineered mechanical properties may not fully recapitulate the complex biomechanical niche of the native meniscus, potentially limiting their efficacy in driving functional tissue regeneration.

Current evidence suggests that HA hydrogels hold therapeutic potential for diverse cartilage-related injuries, including meniscal pathologies, through enhanced tissue preservation and mitigation of inflammation-associated damage. However, traditional articular cartilage repair strategies primarily provide short-term symptomatic relief while failing to regenerate functional hyaline cartilage, leading to repaired tissues and adjacent healthy tissues being susceptible to progressive degeneration and mechanical wear over time (Kowalski et al., 2022). Although the developed HA hydrogel shows promise in restoring compromised cartilage biomechanics and suppressing chondrocyte catabolic activity, critical gaps remain in its clinical translation. Relevant studies lack longitudinal data on hydrogel efficacy under physiological loading environments and have not addressed potential limitations across varying injury severities (e.g., focal defects vs advanced degeneration). Furthermore, the interplay between hydrogel degradation kinetics and sustained biomechanical support in weight-bearing joints remains uncharacterized.

**FIGURE 5:**
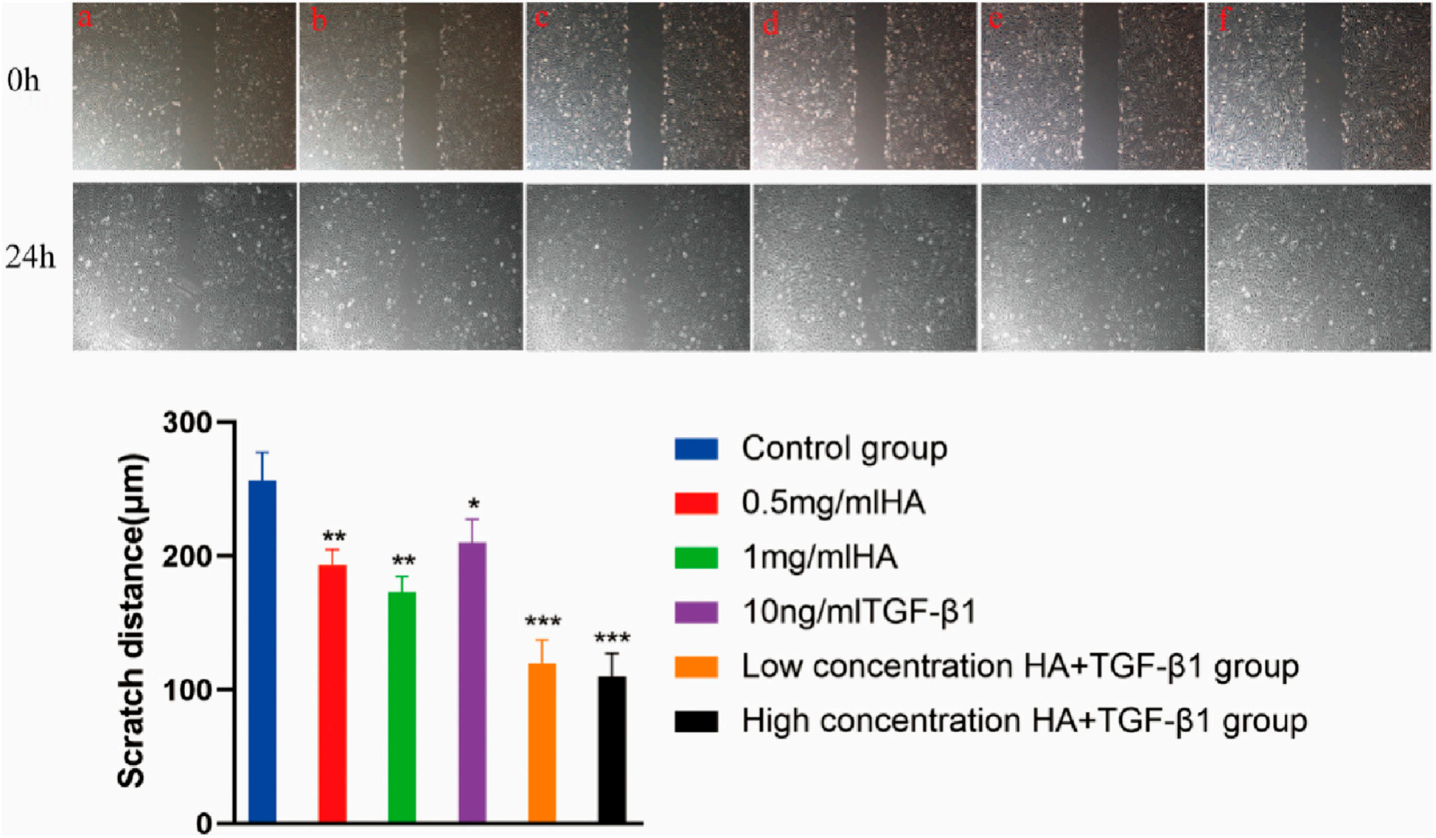
Scratch test image. a Control group; b 0.5 mg/mL HA; c 1 mg/mL HA; d 10 ng/mL TGF-β1; e low concentration HA + TGF-β1; f high concentration HA + TGF-β1. Magnification, Scale bar, 100 µm. Reproduced with permission from ref Wang. et al. (2024b). Copyright^©^ 2024, The Author(s).

**FIGURE 6:**
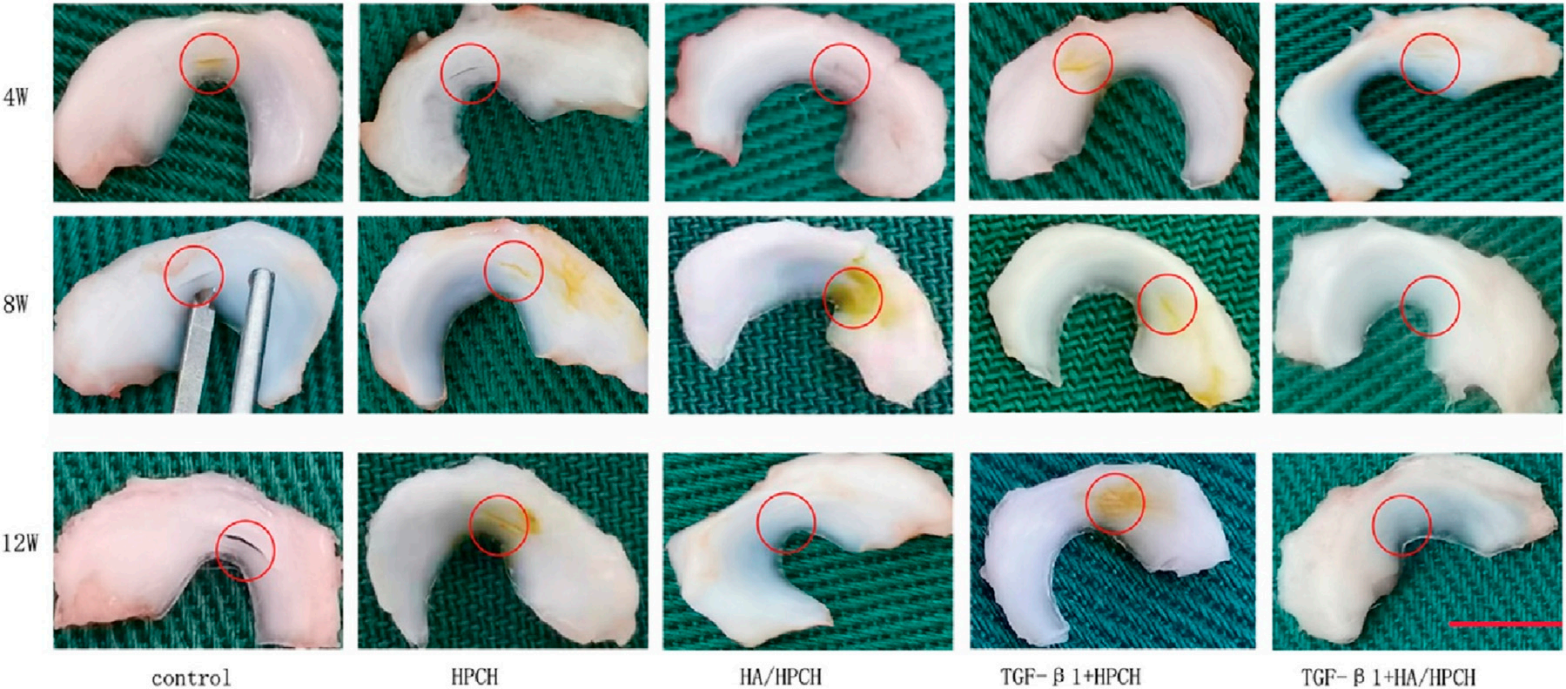
Hyaluronic acid/chitin thermosensitive hydrogel loaded with TGF-β1 promotes meniscus repair in rabbit meniscus full-thickness tear model after **(a)** 4 weeks, **(b)** 8 weeks and **(c)** 12 weeks. Reproduced with permission from ref Wang et al. (2024b). Copyright^©^ 2024, The Author(s).

In animal model validation, the hyaluronic acid/hydroxypropyl chitin (HA/HPCH) thermosensitive hydrogel demonstrated superior efficacy in promoting the repair of full-thickness meniscal tears in a rabbit model (Wang et al., 2024b) compared to other treatment groups, exhibiting excellent temperature sensitivity, biocompatibility, and enhanced capacity to promote cellular proliferation and migration (Figure 5). The study found that the 2% HA-chitin hydrogel containing transforming growth factor β1 (TGF-β1) displayed the highest glycosaminoglycan (GAG) production, indicating its enhanced ability for matrix formation. After injecting this hydrogel into a rabbit model of full-thickness meniscal tears, at 12 weeks post-implantation, the TGF-β1 + HA/HPCH composite hydrogel showed significantly improved meniscal repair outcomes (Figure 6). The newly formed tissue closely resembled normal meniscal tissue in both structural and biochemical characteristics, outperforming other tested hydrogel formulations and control groups.

Another class of promising materials is the polyvinyl alcohol/tannic acid/gelatin/HA (PTGH) hydrogel, which demonstrates favorable mechanical properties and biocompatibility for cartilage tissue engineering applications (Xiang et al., 2023). Although the study primarily focused on articular cartilage repair, the incorporation of HA suggests potential applicability in meniscal injury repair. *In vitro* cell culture results indicated that PTGH hydrogels exert no negative impact on chondrocyte viability and proliferation, supporting their utility in tissue regeneration contexts, including meniscal injury. However, the biocompatibility of gelatin- and hyaluronic acid-based hydrogels is compromised by rapid degradation kinetics, which may diminish their effectiveness as tissue-engineered scaffolds for cartilage repair and regeneration. Additionally, traditional gelatin and HA hydrogels exhibit suboptimal mechanical properties, limiting their application in load-bearing tissues such as articular cartilage.

HA hydrogels themselves can serve as scaffolds for cell-based therapies to promote meniscal injury repair. These hydrogels support cell culture, improve the microenvironment of mesenchymal stem cells (MSCs), and facilitate effective tissue regeneration. Findings from preclinical studies suggest that combining hydrogels with cell-based treatments may improve the outcomes of meniscus repair strategies (Li et al., 2023). The review highlighted significant variability in cell doses used in clinical trials (ranging from 16 × 10^6^ to 150 × 10^6^ cells), which may affect the consistency and comparability of results across studies. The average dose was found to be 41.52 × 10^6^ cells, indicating a lack of standardized dosing protocols that could impact the efficacy of cell-based therapies for meniscus regeneration. The study notes that while various therapeutic strategies, including co-culture systems and composite materials, show promise for meniscus tissue regeneration, the selection of appropriate strategies must be tailored to the specific nature of the injury. This suggests that no universal approach exists, and the complexity of injury patterns may limit the generalizability of preclinical findings to clinical applications.

To further enhance cellular function, one study investigated the effects of meniscal fibrochondrocyte-derived conditioned medium (CM) and transforming growth factor-β3 (TGF-β3) on transduced mesenchymal stem cells (t-MSCs) (Koh et al., 2017), aiming to enhance their chondrogenic potential for effective meniscal tissue engineering. The methodology involved expanding t-MSCs in CM and encapsulating them within a riboflavin-induced photo-crosslinked collagen-hyaluronic acid (COL-RF-HA) hydrogel, which functions as a cell-supportive scaffold to facilitate tissue regeneration. *In vitro* results demonstrated that the combination of CM and TGF-β3 significantly upregulated fibrocartilage-associated gene expression (e.g., COL2, SOX9, ACAN, and COL1) and enhanced the production of extracellular matrix (ECM) components critical for tissue repair. *In vivo* evaluations further revealed that CM-expanded t-MSCs treated with TGF-β3 exhibited optimal performance in cell proliferation, glycosaminoglycan (GAG) accumulation, and collagen deposition, achieving complete regeneration in a meniscal defect model. The study concludes that integrating CM with innovative biomaterial design substantially enhances the chondrogenic differentiation capacity of t-MSCs, thereby promoting meniscal regeneration. This research provides a promising approach to improving meniscal repair strategies, with the potential to improve clinical outcomes for patients with meniscal injuries. However, it should be noted that although the study demonstrated the effects of conditioned medium (CM) and transforming growth factor-β3 (TGF-β3) on transduced mesenchymal stem cells (t-MSCs), it provided limited insights into the molecular-level interaction mechanisms of these factors. The long-term implications of this therapeutic approach, including the durability of regenerated tissue and potential late-onset complications, remain unassessed, constraining the understanding of the proposed method’s long-term efficacy and safety. Furthermore, no direct comparative analysis was conducted between the proposed strategy and established meniscus repair techniques, such as allografts or synthetic implants.

## 4 Clinical application research

HA hydrogels, distinguished by their exceptional biocompatibility, tunable mechanical properties, and ability to promote tissue regeneration, have emerged as highly promising ideal materials for meniscus repair, with significant progress also achieved in related clinical applications. Taking Hymovis^®^ (manufactured by Fidia Farmaceutici SPA) as an example, it is a hexadecylamide derivative of hyaluronic acid, formulated as a sterile, pyrogen-free, viscoelastic hydrogel for intra-articular injection. It has been used for treating patients with symptomatic knee osteoarthritis (Pavelka et al., 2020), hip osteoarthritis (Migliore et al., 2020), and meniscal lesions (Zorzi et al., 2014). Clinical trials have substantiated the positive clinical efficacy of such viscosupplements in patients undergoing arthroscopic partial meniscectomy. Studies have demonstrated that intra-articular injection of Hymovis^®^ effectively alleviates patient pain, improves joint mobility, and reduces the use of non-steroidal anti-inflammatory drugs (NSAIDs). The specific advances of Hymovis^®^ in the clinical application of meniscus repair will be detailed in the following section.

### 4.1 clinical efficacy evaluation

The clinical efficacy of HA hydrogels in meniscus repair is reflected in improved symptoms, enhanced tissue regeneration, and restored joint function. Clinical studies demonstrate that intra-articular HA hydrogel injections significantly reduce pain, improve joint mobility, and enhance patients’ quality of life (Zorzi et al., 2014). Supporting this, Zorzi et al. recruited 50 participants with degenerative meniscal tears and conducted a single-centre, observer-blinded, parallel-group clinical study to investigate the efficacy of Hymovis^®^ in the management of meniscal tears and meniscal tear repair (Zorzi et al., 2015). Clinical outcomes included pain reduction (Visual Analogue Scale), improved knee function (WOMAC questionnaire), reduced meniscal tear length and depth (MRI-confirmed), and SF-36 questionnaire scores. Clinical assessments were conducted at baseline, as well as on days 14, 30, and 60, to evaluate multiple outcome measures. Results showed that the HYADD4^®^ group exhibited significant pain reduction by day 14 (p < 0.001), with sustained improvements in subsequent follow-ups. Compared to the control group, the HYADD4^®^ group demonstrated significant reductions in meniscal lesion length and depth (p < 0.001). These findings suggest that HYADD4^®^ may represent a novel therapeutic option for the conservative management of patients with painful meniscal tears. MRI data further indicated that this hydrogel formulation might actively contribute to the healing process of meniscal lesions. In conclusion, HYADD4^®^ significantly alleviated pain, improved knee function, and promoted meniscal lesion repair through iA administration, offering a potentially effective treatment strategy for meniscal tear patients. ​​However, limitations of this study include the involvement of a limited number of participants, where the small sample size may compromise the reliability of conclusions. The relatively short follow-up period restricted the ability to assess long-term therapeutic efficacy and safety. Despite demonstrating clinical benefits, the study did not explore the biological mechanisms underlying the observed improvements.

Knee osteoarthritis (KOA) and meniscal tears (MT) are two of the most common knee injuries, significantly impacting patients’ quality of life (QoL). A non-interventional, prospective, multicenter study involving 165 patients with KOA and/or MT evaluated the effects of intra-articular Hymovis^®^ injection on QoL, physical mobility, and satisfaction levels in patients with isolated KOA, isolated MT, or both conditions (Balius et al., 2023). Results demonstrated significant QoL improvement (>80%) across all three patient groups, with statistically significant improvements (p < 0.001) in both KOOS and WOMAC scores compared to baseline; post-treatment enhancements were observed in physical activity levels, sports and recreational capacity, and treatment satisfaction (p < 0.001), while pain symptoms also significantly improved (p < 0.05) across all groups, collectively demonstrating that Hymovis^®^ intra-articular injection improves quality of life, physical function, and clinical outcomes in patients with KOA, MT, or both comorbidities.

Degenerative meniscus lesion (DML) presents in adult patients (35–65 years of age) who have not had a trauma and consists in a progressive delamination and surface fibrillation. Bertondenet et al. evaluated the clinical efficacy and healing effects of conservative management of DMLs with Hymovis^®^ (Berton et al., 2020). Significant improvements were observed between baseline and follow-up in WOMAC scores, physical function, patient global assessment (ptGA), clinician global assessment (CoGA), and SF-36 indices (Lam et al., 2014). The treatment demonstrated good tolerability, with only one patient requiring further surgical intervention (arthroscopic partial meniscectomy) 1 year post-treatment. This study supports the use of HA in the conservative management of DML, as evidenced by T2 measurements in MRI scans, indicating HA’s clinical efficacy in enhancing meniscal healing processes. A major limitation highlighted is the lack of evaluation correlating treatment outcomes with the degree of meniscal degeneration. HA’s therapeutic effects may vary depending on the severity of degeneration, and the high degenerative status of enrolled patients may influence the observed results.

### 4.2 Safety

Hyaluronan-based hydrogels demonstrate good safety and tolerance profiles, with a low incidence of adverse events reported in clinical applications (Migliore et al., 2009; Humphries et al., 2025). This is supported by multiple meta-analyses that have evaluated the differences in safety between intra-articular hyaluronic acid (IAHA) and intra-articular placebo control (Bellamy et al., 2006; Bannuru et al., 2015). A Cochrane review encompassing 76 randomized controlled trials (RCTs) was unable to draw definitive conclusions regarding the safety of HA products due to sample size limitations; however, it found no serious safety concerns. Furthermore, IAHA demonstrated efficacy comparable to systemic therapies (such as oral NSAIDs), though with a higher incidence of local reactions and a lower incidence of systemic adverse events (Bellamy et al., 2006). Reinforcing this view, multiple other meta-analyses also consistently conclude that HA demonstrates good tolerance, with a low incidence of adverse events and an absence of serious risk (Strand et al., 2015; Bannuru et al., 2016a; Bannuru et al., 2016b). However, it is noteworthy that most previous meta-analyses on the safety of IAHA relied solely on published data, where underreporting of safety data is common. Furthermore, these analyses did not adequately account for the concurrent use of oral non-steroidal anti-inflammatory drugs (NSAIDs) by patients in some clinical trials. Both factors may have compromised the accuracy of the safety assessments. Additionally, a contrasting finding comes from a 2012 meta-analysis which reported that IAHA may be associated with an increased risk of serious adverse events (Rutjes et al., 2012).

### 4.3 Combined therapeutic strategies

The combined application of hyaluronan-based hydrogels with other therapeutic approaches demonstrates synergistic effects in meniscus injury repair, leading to further enhanced treatment outcomes. Firstly, hyaluronan hydrogels serve as effective carriers for stem cells, promoting their colonization and differentiation at the injury site, thereby augmenting tissue regeneration. For example, studies indicate that combined therapy utilizing hyaluronan hydrogels and mesenchymal stem cells (MSCs) significantly improves the regenerative capacity and functional recovery of meniscal tissue (Rhim et al., 2021). Secondly, hyaluronan hydrogels possess the ability to adsorb and provide sustained release of bioactive factors, such as transforming growth factor-beta (TGF-β) and fibroblast growth factor-2 (FGF-2), enhancing their biological activity. Illustrating this, combination therapy employing hyaluronan hydrogels with TGF-β has been shown to significantly promote the synthesis and remodeling of the meniscal ECM (Ding et al., 2022). Thirdly, hyaluronan hydrogel treatment combined with physical therapy modalities, including functional exercises and electrical stimulation, can further improve joint function and alleviate pain. For instance, clinical research demonstrates that the combination of hyaluronan hydrogels and physical therapy significantly enhances patients’ knee joint functional scores and quality of life (Onu et al., 2024).

## 5 Limitations and future perspectives

Despite demonstrating significant potential in meniscal injury repair, current research on hyaluronan-based hydrogels faces limitations in three key aspects: material properties, clinical study design, and scope of application. Firstly, insufficient mechanical strength (as mentioned in section 2.2.2), uncontrolled degradation rates, and functional simplicity hinder their ability to meet the demands of complex injury repair. Secondly, clinical studies are commonly constrained by small sample sizes, short follow-up periods, and a lack of rigorous control groups, compromising the reliability and generalizability of findings. Finally, their applicability remains limited primarily to mild-to-moderate injuries, with further restrictions imposed by individual variability and high costs, presenting barriers to personalized treatment and widespread clinical adoption. Therefore, the development of personalised treatment and the clinical translation of HA hydrogels in this field still face two core obstacles. One of these challenges stems from the complex anatomical structure and dynamic biological environment of the meniscus. The precise dimensions, curvature, and mechanical gradients of the injured area must be customised using high-precision MRI data, but the degradation rate of the hydrogel is difficult to match the highly variable concentrations of synovial inflammatory factors in individual patients. Additionally, biofunctional customisation faces a dilemma: high cross-linking density can bear weight but inhibits cell migration, while low cross-linking promotes regeneration but may cause rapid collapse. The practical requirement for low viscosity during arthroscopic injection further complicates the balance of material properties. On the other hand, cost and manufacturing bottlenecks permeate the entire chain from R&D to payment. Personalised manufacturing relies on expensive raw materials and processes, with regulatory and clinical translation costs being particularly prominent. Long-term MRI follow-up and cold chain transportation continue to drive up expenses.

Future research should prioritize optimizing the mechanical properties, degradation kinetics, and functional diversity of hyaluronan hydrogels; conducting large-scale, multi-center randomized controlled trials (RCTs) with extended follow-up to comprehensively evaluate efficacy and safety; and exploring their potential for complex injuries while integrating complementary therapeutic approaches to enhance repair outcomes. Additionally, refining manufacturing processes to reduce costs is essential to facilitate broader clinical implementation, thereby enabling safe and effective treatment for more patients.

Looking further ahead, future development directions for hyaluronan hydrogels in meniscal repair encompass three domains: 1) Materials science innovation (developing smart hydrogels responsive to pH/temperature/enzymes, functionalizing with cell-adhesive peptides or growth factor binding sites, and creating composites with collagen/chitosan to enhance mechanical strength, bioactivity, and multifunctionality); 2) Personalized medicine strategies (designing precision treatment plans based on individual patient profiles, utilizing biomarkers to assess injury severity and repair progression, and developing tailored delivery systems); and 3) Novel technologies/methods (leveraging gene editing like CRISPR-Cas9 to modulate cellular regeneration, integrating mesenchymal stem cells (MSCs) into hydrogel composite systems, and employing advanced imaging such as MRI for real-time repair monitoring). These innovations will drive efficient and precise applications of hyaluronan hydrogels, offering safer and more personalized therapeutic solutions.

## 6 Conclusion

Hyaluronan-based hydrogels exhibit substantial promise for meniscal injury repair and cell culture applications. Their capacity to mimic the native ECM, support cellular growth and differentiation, and deliver therapeutic agents establishes them as invaluable tools in tissue engineering. Despite persistent challenges, ongoing research and advancements in hydrogel technology hold significant potential to overcome current limitations, paving the way for clinical translation and widespread adoption in meniscal repair.
